# Curcumin represses lipid accumulation through inhibiting ERK1/2-PPAR-γ signaling pathway and triggering apoptosis in porcine subcutaneous preadipocytes

**DOI:** 10.5713/ab.21.0371

**Published:** 2021-11-01

**Authors:** Shifeng Pan, Yongfang Chen, Lin Zhang, Zhuang Liu, Xingyu Xu, Hua Xing

**Affiliations:** 1 College of Veterinary Medicine, Yangzhou University, Yangzhou, Jiangsu, 225009, China; 2 Jiangsu Co-Innovation Center for the Prevention and Control of Important Animal Infectious Disease and Zoonoses, Yangzhou, Jiangsu, 225009, China; 3 Department of Animal Science, Washington State University, Pullman, WA 99163, USA

**Keywords:** Adipogenic Differentiation, AKT/BCL-2/BAX Signaling Pathway, Apoptosis, Curcumin, ERK1/2-PPAR-γ Signaling Pathway, Porcine Subcutaneous Preadipocytes

## Abstract

**Objective:**

Excessive lipid accumulation in adipocytes results in prevalence of obesity and metabolic syndrome. Curcumin (CUR), a naturally phenolic active ingredient, has been shown to have lipid-lowering effects. However, its underlying mechanisms have remained largely unknown. Therefore, the study aims to determine the effect of CUR on cellular lipid accumulation in porcine subcutaneous preadipocytes (PSPA) and to clarify novel mechanisms.

**Methods:**

The PSPA were cultured and treated with or without CUR. Both cell counting Kit-8 and lactate dehydrogenase release assays were used to examine cytotoxicity. Intracellular lipid contents were measured by oil-red-o staining extraction and triglyceride quantification. Apoptosis was determined by flow cytometry and the terminal deoxynucleotidyl transferase-mediated deoxyuridine triphosphate-nick end labelling assay. Adipogenic and apoptosis genes were analyzed by quantitative polymerase chain reaction and Western blot.

**Results:**

The CUR dose-dependently reduced the proliferation and lipid accumulation of PSPA. Noncytotoxic doses of CUR (10 to 20 μM) significantly inhibited extracellular signal-regulated kinase 1/2 (ERK1/2) phosphorylation and expression of adipogenic genes peroxisome proliferation-activity receptor-γ (PPAR-γ), CCAAT/enhancer binding protein-α, sterol regulatory element-binding protein-1c, adipocyte protein-2, glucose transporter-4 as well as key lipogenic enzymes fatty acid synthase and acetyl-CoA carboxylase, while ERK1/2 activation significantly reversed CUR-reduced lipid accumulation by increasing PPAR-γ. Furthermore, compared with differentiation induced media treated cells, higher dose of CUR (30 μM) significantly decreased the expression of AKT and B-cell lymphoma-2 (BCL-2), while increased the expression of BCL-2-associated X (BAX) and the BAX/BCL-2 expression ratio, suggesting triggered apoptosis by inactivating AKT and increasing BAX/BCL-2 ratio and Caspase-3 expression. Moreover, AKT activation significantly rescued CUR inhibiting lipid accumulation via repressing apoptosis.

**Conclusion:**

These results demonstrate that CUR is capable of suppressing differentiation by inhibiting ERK1/2-PPAR-γ signaling pathway and triggering apoptosis via decreasing AKT and subsequently increasing BAX/BCL-2 ratio and Caspase-3, suggesting that CUR provides an important method for the reduction of porcine body fat, as well as the prevention and treatment of human obesity.

## INTRODUCTION

Nowadays, obesity is increasingly recognised as a significant risk for multiple metabolic syndrome, such as hyperlipidemia, type 2 diabetes, hypertension, cardiovascular diseases and diverse cancers [1]. Therefore, obesity is considered as a global health problem [2]. Numerous evidence shows that the expansion of adipose tissue during the development of obesity largely depends on adipocytes hyperplasia (abnormal increase in the number) and hypertrophy (an increase in the size) [3], the former of which is closely associated with increased adipogenic differentiation and preadipocytes proliferation, whereas the latter is related to the lipolysis of accumulated intracellular lipids and/or apoptosis of mature adipocytes [4]. Both decreased adipogenesis and increased lipid catabolism are contributed to reduced lipid accumulation. Therefore, controlling adipogenesis plays a vital role in decreasing the fat mass and stopping obesity progression.

Adipogenesis is a multistep process governed by a com plex and sequential transcriptional activation of numerous adipogenic genes [5]. Among which, peroxisome proliferator-activated receptor-γ (PPAR-γ), CCAAT/enhancer-binding protein-α (C/EBP-α), and sterol regulatory element-binding protein-1c (SREBP-1c), are primary transcription factors contributing to differentiation and *de-novo* lipogenesis through activating key lipogenic enzymes fatty acid synthesis (FAS) and acetyl-CoA carboxylase (ACC). Previous studies showed that the extracellular signal-regulated kinases 1/2 (ERK1/2) signaling pathway plays a key role in adipocyte proliferation and differentiation by targeting above adipogenic genes [6]. A number of studies demonstrated that ERK1/2 activation promoted adipogenic differentiation [7], while others showed that ERK1/2 signaling pathway played a negative role in regulating adipogenic differentiation and lipid accumulation [8], these conflicting results indicated further studies should be conducted to clarify it’s role in adipogenic differentiation.

Studies confirmed that the decrease in the number of mature adipocytes induced by apoptosis activation was a contributing factor to body fat mass reduction [9], thus, the trigger of mature adipocytes apoptosis has attracted greater attention as a potentially promising approach in preventing obesity and related metabolic diseases. The Bcl-2 family proteins are key regulators of apoptosis, which include BAX (pro-apoptotic protein) and Bcl-2 (anti-apoptotic protein). Apoptosis is initiated by activating either intrinsic or extrinsic pathways and leads to the activation of Caspase-3 [10], the executor of apoptosis. Previous studies showed that inactivation of serine-threonine kinase AKT is able to activate apoptosis by increasing the expression of BAX, and thus participate in diverse biological regulations [11]. However, whether AKT signaling pathway mediated apoptosis can be involved in the regulation of adipogenic differentiation in porcine subcutaneous preadipocytes (PSPA) has not been well clarified.

In past decades, although antiobesity approaches based on surgical options and pharmaceutical interventions have been appeared to be effective in fat mass control [12], the serious adverse effects obtained with these drugs should not be ignored. So far, antiobesity drugs that have been developed have limited efficacies and considerable adverse effects, so until now only a few drugs are admitted for potential antiobesity treatment. Therefore, there is an urgent need to find much safer and more effective antiobesity strategies. Under these circumstances, through dietary modifications, the non pharmacological approach, is becoming the primary strategy [13]. Moreover, due to the positive efficacy and minimal side effects, increased attention has been focusing on natural active ingredients of plant origin, which have been reported as alternative therapeutic agents to reduce body fat mass and protect against the development of obesity.

Curcumin (CUR), a natural active substance extracted from turmeric plants of the ginger family, has exhibited several beneficial properties, especially a lipid-lowering effect [13], therefore it has aroused widespread interest in biological medicine. Accumulating evidence in high-fat diet (HFD) induced obese mice has demonstrated preventive effects of CUR in controlling fat mass [14], showing anti-obesity potential. Moreover, *in vitro* studies using 3T3-L1 cell line also demonstrated inhibitory effect of CUR on adipogenic differentiation. However, so far, there is no report concerning the effect of CUR on adipogenic differentiation of PSPA. Moreover, the potential mechanism underlying has yet to be elucidated. Especially, whether ERK1/2-PPAR-γ signaling pathway and apoptosis are involved in CUR regulated lipid accumulation of PSPA needs to be further studied.

## MATERIALS AND METHODS

### Ethics statements

This study was carried out in strict accordance with the recommendations in the Guide for the Care and Use of Laboratory Animals of the Ministry of Science and Technology of the People’s Republic of China. The protocols for animal experiments were approved by the Jiangsu Administrative Committee for Laboratory Animals (Approval number: SYXK-SU-2007-0005), and complied with the guidelines of Jiangsu laboratory animal welfare and ethics of Yangzhou University Animal Care and Use Committee, China.

### Reagents

Curcumin (purity over 99%), dexamethasone, 3-isobutyl-1-methylxanthine (IBMX), insulin, and annexin V-fluorescein isothiocyanate/propidium iodide (V-FITC/PI) apoptosis detection kits were purchased from Sigma-Aldrich (St Louis, MO, USA). Dulbecco’s modified eagle medium: Nutrient Mixture F-12 (DMEM/F12), fetal bovine serum (FBS), Type I collagenase, penicillin and streptomycin were obtained from Gibco (Waltham, MA, USA). Triglyceride (TG) determination kit, glucose oxidase assay kit and glucose-6-phosphate dehydrogenase (GPDH) activity measurement kit were gained from Applygen Technologies (Beijing, China). Cell counting kit-8 (CCK-8), lactate dehydrogenase (LDH) release kit and oil red O (ORO) were provided by Nanjing Jiancheng Bioengineering Insititute (Nanjing, China).

### Cell culture

Six male Meishan piglets (a typical Chinese indigenous obese breed) at weaning stage (35 days old) used in the present study were obtained from the Breeding Center of Small Meishan Pigs (Jurong, Jiangsu, China). All the piglets were sacrificed by exsanguination after electrical stunning and the subcutaneous fat tissue (SAT) was rapidly sampled from the neck and back. PSPA were isolated and pooled together according to our published protocols [15,16]. Briefly, SAT samples were rinsed with DMEM (containing 100 IU/mL penicillin/streptomycin) without FBS, which were then cut into small pieces and digested at 37°C water bath with digestive fluid (DMEM plus 20 g/L bovine serum albumin [BSA], 1 g/L type IV collagenase) for nearly 1 h. Then, DMEM with 10% FBS was used to stop digestion. The suspension was respectively filtered by 150, 75, 38, and 23 μm pore sizes sterile nylon filters to remove undigested tissues, and then the filtrate was centrifuged at 1,000 rpm for 10 min to get the PSPA pellet. After erythrocyte lysis [15], the PSPA was then cultured in DMEM (containing 10% FBS, 100 IU/mL penicillin/streptomycin and 2 mM L-glutamine) (Invitrogen, Carlsbad, CA,USA) at a density of 3×10^4^ cells/cm^2^ in a humidified atmosphere of 5% CO_2_ at 37°C.

### Adipogenic differentiation of PSPA

On day 3 after 85% to 90% confluence (day 0), PSPA were divided randomly into 0 (only dimethyl sulfoxide, DMSO), 10, 15, 20, and 30 μM CUR groups. Meanwhile, these five groups were all exposed to differentiation induced media (DIM, 10% FBS DMEM containing 0.25 μM dexamethasone, 0.25 mM IBMX, and 1 μg/mL insulin). PSPA treated with only DMSO was used as negative control (control). PSPA were harvested after 48 h of CUR treatment for further detection of the expression of adipogenic genes.

### Cell viability assay

PSPA were seeded at a density of 1×10^3^ cells, and cultured with 100 μL DMEM with 10% FBS. After 48 h treatment of CUR,10 μL CCK-8 reagent was added per well and were cultivated at 37°C with 5% CO_2_ incubator for another 2 h. The optical density at 450 nm (OD450) were detected by a microplate reader (Synergy HTX, BioTek, Winooski, VT, USA).

### Lactate dehydrogenase assay

LDH release assay kit was used to evaluate LDH release from cells after CUR exposure according to the manufacturer’s protocol. Both media and cell lysates LDH activities were measured by absorbance at 440 nm. Cytotoxicity index is calculated as LDH release into media/total LDH (LDH in both media and cell lysates). Data are expressed as a percentage of LDH in CUR-treated cells relative to that in DIM cells. The percentage of viable cells was calculated by defining the cell viability without treatment as 100%.

### Lipid quantification

The ORO staining extraction was employed to measure the lipid content. PSPA were fixed with 10% formalin for 5 min at room temperature after washed with phosphate-buffered saline (PBS) for three times. Then PSPA were stained with ORO working solution (0.2% ORO in 60% isopropanol). ORO staining was determined using a modified protocol. The PSPA were washed twice with PBS, fixed with 10% formalin for 1 h, dried for 10 min, and stained with ORO for 10 min. The PSPA were sequentially washed with 70% ethanol and water and were then dried. The ORO stained lipid droplets were visualized by microscopy (OlympusIX73, Tokyo, Japan), which were dissolved in isopropanol and quantified by measuring absorbance at 510 nm with a microplate reader (SynergyTM HTX, BioTek, Winooski, VT, USA).

### Intracellular triglyceride content

After exposure to DIM with or without CUR for 48 h, PSPA were washed with PBS for 3 times, and were then lysised in the ice with adequate RIPA for 20 min. Then PSPA were collected and sonicated in a triacylglycerol assay buffer containing 1 mM ethylenediaminetetraacetic acid (EDTA) and 50 mM Tris-HCl (pH 7.4), then centrifuged for 2 min (4°C, 12,000 ×g). Coomassie brilliant blue method was used for protein quantification with 50 μL supernatant. The remaining supernatant was blended with 2 times volume of methanol-chloroform mixture (v/v = 1/1) and stood for 30 min, then centrifuged for 2 min (4°C, 12,000×g), discarded the upper solution and dried, then added 15 μL PBS to dissolve lipid. A colorimetric triacylglycerol kit (Nanjing Jiancheng Bioengineering Institute, NanJing, China) were used for detecting triacylgycerol levels.

### Glucose consumption determination

Glucose consumption was performed by glucose oxidase assay kit. The media samples were collected to 1.5 mL centrifuge tubes. Glucose content was normalized with total protein that was detected by the bicinchoninic acid (BCA) assay (Thermo Fisher Scientific, Waltham, MA, USA).

### GPDH activity assay

A GPDH activity assay kit was used to measure the GPDH activity, by determining the decrease of dihydronicotinamide-adenine dinucleotide during GPDH-catalyzed reduction of dihydroxyacetone phosphate. The differentiated adipocytes were firstly rinsed with PBS, and then were scraped into enzyme extraction buffer and sonicated. After centrifugation (10,000 rpm, 5 min), GPDH activity in media was measured by a microplate readerat 340 nm. The GPDH assay results were normalized with protein concentration. The percentage of GPDH activity in CUR-treated cells was calculated relative to DIM cells.

### Cell apoptosis assay-annexin V-FITC and PI staining

After treatment with 30 μM CUR for 48 h, 5 μL of annexin V-FITC and 5 μL PI were used to stain the cells for 10 min at room temperature. Cells were then subjected to fluorescence-activated cell sorting analys is using a flow cytometer (Arla BD, USA), with apoptotic cells being annexin V-positive/PI-negative.

### RNA extraction and real-time quantitative polymerase chain reaction

Total RNA was extracted by TRIzol total RNA extraction kit (Invitrogen Life Technologies, Carlsbad, CA, USA) and reverse transcribed into cDNA by a PrimeScript First Strand cDNA Synthesis kit (no. D6110A, Takara Biotechnology Co. Ltd., Dalian, China). Quantitative reverse-transcription polymerase chain reaction (qRT-PCR) was performed by Applied Biosystems 7500 Real-Time PCR system (Thermo Fisher Scientific, Inc., USA) with 2 μL 1: 20 diluted cDNA, and gene expression was normalized to peptidylprolyl isomerase A (PPIA), after comparing with GAPDH and β-actin through NormFinder analysis. The PCR amplification was carried out by denaturation at 94°C for 25 s followed by 40 cycles of annealing (62°C for 40 s) and extension. Relative gene expression was determined using the 2^−ΔΔ^Ct method. All primers sequences used in the current study are shown in Table 1 and were synthesized by Genewiz, Inc (Suzhou, China).

### Western blot

Total protein was extracted using RIPA lysis buffer (Thermo Fisher Scientific, Inc., USA), containing 2 mM EDTA, 100 mM NaCl, 5% sodium dodecyl sulfate (SDS), 50 mM NaF, 0.1 mM Na_3_VO4, 100 μM 4-(2-aminoethyl) benzenesulfonyl fluoride hydrochloride, 1 mM benzamidine, 50 mM 4-(2-hydroxyethyl)-1-piperazineethanesulfonic acid (pH 7.4) and 10 μg/mL aprotinin. Protein concentration was quantified using the BCA Protein Assay Kit. A total of 40 μg protein extract was mixed with loading buffer and denatured for 5 min by boiling before being loaded onto a 10% SDS-PAGE gels. The proteins were separated by electrophoresis, electrotransferred onto nitrocellulose membranes (EMD Millipore, Billerica, MA, USA), and blocked for 90 min at room temperature using tris-buffered saline (TBS)-Tween-20 (TBST) (J&K Scientific, Ltd., Shanghai, China) containing 3% BSA. Subsequently, membranes were probed overnight at 4°C with antibodies directed against PPAR-γ, C/EBP-α, SREBP-1c, AP2, FAS, ACC, GLUT4, ERK1/2, p-ERK1/2, AKT, and p-AKT in TBST. After incubation with goat anti-rabbit immunoglobulin G peroxidase-conjugated secondary antibodies (Bioworld Technology, St Louis Park, MN, USA) at a dilution of 1:10,000, proteins were visualized using an enhanced chemiluminescence with the LumiGlo substrate (Super Signal West Pico Trial Kit, Pierce, Rockford, IL, USA), the bands were visualized and captured by VersaDoc 4000MP system (Bio-Rad, Hercules, CA, USA), and then Quantity One software (Bio-Rad, USA) was used to calculate the value of band density automatically. Protein expression levels were showed as relative-fold change of the DIM cells. β-Actin was used as a reference protein. The information of antibodies used in the study are listed in Table 2.

### Statistical analysis

All data are shown as the mean±standard error of the means. Statistical Program for Social Sciences (SPSS) software 20.0 for Windows (SPSS Inc., Chicago, IL, USA) were used to carry out the statistical analyses. The differences were tested with the one-way analysis of variance, followed by Duncan’s multiple comparisons test. A p-value of less than 0.05 was considered significant. All the experiments were repeated at least three times.

## RESULTS

### Curcumin dose-dependently inhibited the cell viability and increased the cytotoxicity

**Figure 1:**
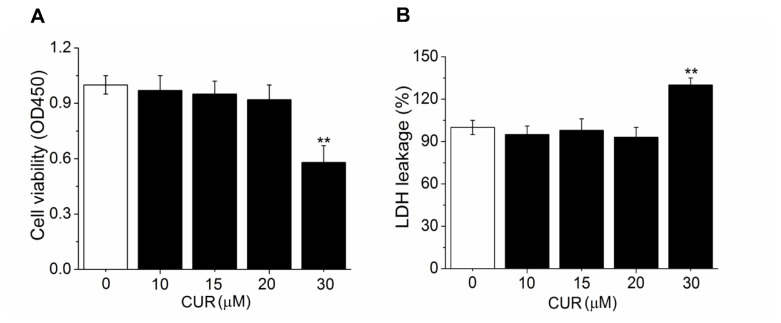
Effect of CUR on cell viability and cytotoxicity. (A) The cell viability measured by CCK assay. (B) The cytotoxicity detected by LDH leakage. All values are presented as mean±standard error of the mean, n = 6. CUR, curcumin; CCK, cell counting kit; LDH, lactate dehydrogenase. ** p<0.01.

Based on the possibility that CUR may cause cytotoxicity to PSPA, the cell viability and cytotoxicity were measured by CCK-8 and LDH release assay. Results showed that compared with DIM cells, with the increase of CUR doses, the cell viability was gradually inhibited (approximately 42% lower in 30 μM CUR treated cells than that in DIM cells) (Figure 1A). Furthermore, low doses of CUR (10 to 20 μM) showed no change on LDH leakage, while higher dose of CUR (30 μM) presented significant elevated LDH release (Figure 1B), indicating that CUR dose-dependently enhanced the cytotoxicity.

### Curcumin dose-dependently reduced intracellular triglyceride content and lipid accumulation

**Figure 2:**
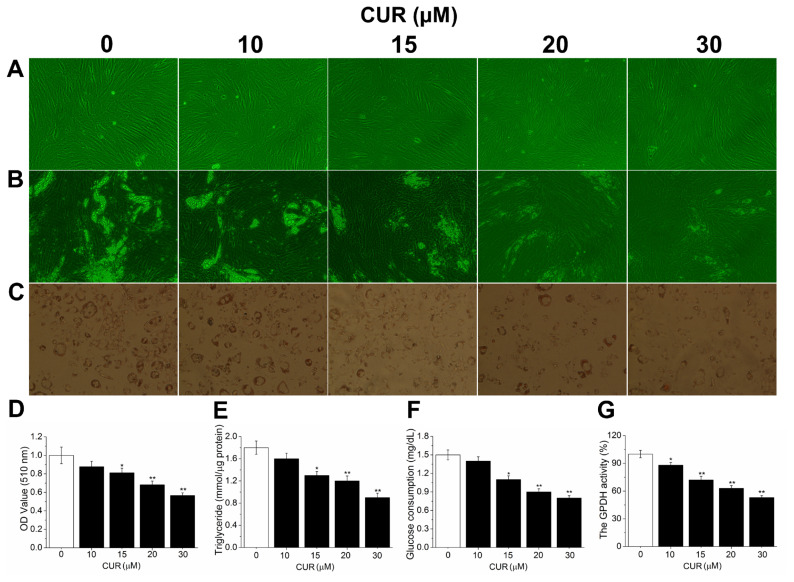
Effect of CUR on intracellular lipid accumulation. (A) PSPA reaches 85% to 90% confluence (100×). (B) Intracellular lipid accumulation (100×). (C) ORO-stained intracellular lipids in adipocytes (100×). (D) ORO extracted and quantified by measuring absorbance at 510 nm. (E) Intracellular TG content. (F) Glucose consumption. (G) The GPDH activity. All values are presented as mean±standard error of the mean, n = 6. CUR, curcumin; PSPA, porcine subcutaneous preadipocytes; ORO, oil red O; TG, triglyceride; GPDH, glucose-6-phosphate dehydrogenase. * p<0.05 and ** p<0.01.

To explore the role of CUR inreducing intracellular TG content and lipid accumulation, differentiation of PSPA was induced and then they were incubated with different doses of CUR after 85% to 90% confluence (Figure 2A). Lipid accumulation was observed under light microscopy, while intracellular TG content was quantified by ORO staining extraction and TG quantification. Results showed that compared with DIM cells, with the increasing dose of CUR, the amount of lipid droplets was gradually decreased (Figure 2B). ORO staining showed that the positive rate of cytoplasmic ORO staining was obviously decreased (Figure 2C). Isopropanol extraction result showed that lipid droplets stained with ORO were reduced by 12.3%, 18.9% (p<0.05), 31.8% (p<0.01), and 43.5% (p<0.01) after exposure to 10, 15, 20, or 30 μM CUR, respectively (Figure 2D). Furthermore, the TG quantification assay result also confirmed the observed decrease in lipid accumulation (Figure 2E), suggesting reduced lipid accumulation by CUR. In addition, CUR treated PSPA consumed less glucose compared with DIM cells (Figure 2F). Meanwhile, the activity of GPDH, a key enzyme in the lipid biosynthesis, was also obviously suppressed compared with DIM cells (Figure 2G). Collectively, these results suggested that CUR dose-dependently inhibited intracellular TG content and lipid accumulation. Specifically, 20 μM CUR presented the most obvious inhibitory effect on adipogenic differentiation without cytotoxicity.

### Curcumin significantly inhibited the expression of key adipocytes regulatory genes

**Figure 3:**
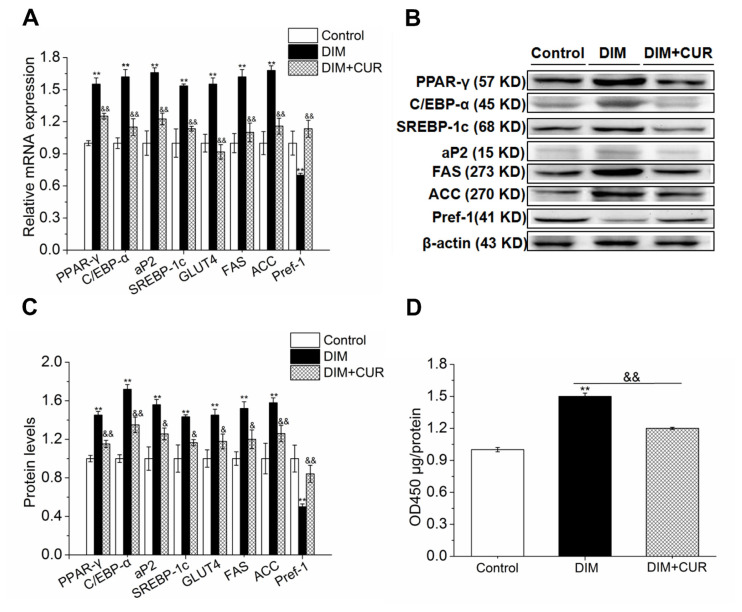
Effect of CUR on the expression levels of adipogenic genes and PPAR-γ transcriptional activity. (A–C) The expression levels of adipogenic genes. (D) The PPAR-γ transcriptional activity. All vaules are presented as the mean±standard error of the mean, n = 6. CUR, curcumin; PPAR-γ, peroxisome proliferation-activity receptor-γ; DIM, differentiation induced media. ** p<0.01, compared with control cells; ^&^ p<0.05 and ^&&^ p<0.01, compared with DIM cells.

To further clarify whether the inhibitory effect of CUR on lipid accumulation is accompanied by inhibiting adipogenesis and lipogenesis, the mRNA and protein levels of several key adipocytes regulatory genes were measured. As illustrated in Figure 2, 20 μM CUR maximally inhibited the intracellular TG content and lipid accumulation without cytotoxicity, therefore in the further study, only DIM and 20 μM CUR treated cells were used to evaluate the expression of adipogenic genes. Results showed that 20 μM CUR significantly reduced C/EBP-α, PPAR-γ, SREBP-1c, AP2, and GLUT4 expression levels compared with DIM cells. In addition, the expression of FAS and ACC, two key lipogenetic enzymes that are essential for *de novo* lipogenesis, were both significantly inhibited (p<0.05). However, the expression of preadipocyte factor-1 (Pref-1), which exerts inhibitory action on adipogenesis and was shown as an excellent preadipocytes marker, was significantly enhanced (p<0.01) in 20 μM CUR cells compared with DM cells (Figure 3A–C). These results indicate that the inhibitory effect of CUR on lipid accumulation is associated with the inhibition of adipogenesis and lipogenesis.

Since we have showed that CUR significantly inhibited PPAR-γ expression, we further determined the PPAR-γ transcriptional activity. In Figure 3D, CUR significantly inhibited PPAR-γ transcriptional activity, which was consistent with decreased expression of PPAR-γ, further confirmed that CUR inhibited intracellular lipid accumulation by PPAR-γ suppression.

### Curcumin suppressed adipogenic differentiation and intracellular lipid accumulation by repressing ERK1/2-PPAR-γ signaling pathway

**Figure 4:**
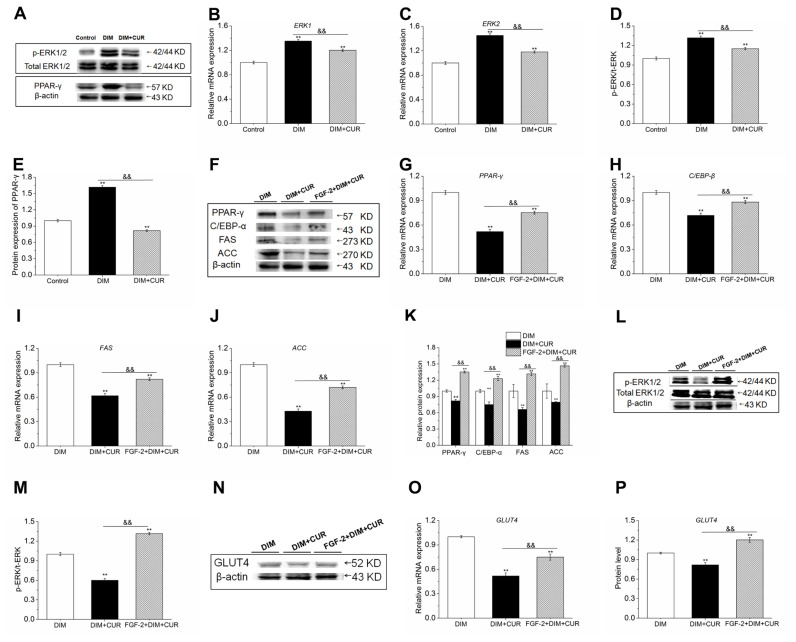
Effect of CUR on ERK1/2-PPAR-γ signaling pathway and the reverse effect of ERK activation on CUR inhibited adipogenic genes. (A–B) The mRNA expression of ERK1 and ERK2. (C–D) The phosphorylation of ERK1/2. (E) The protein level of PPAR-γ. (F–K) Expression levels of PPAR-γ, C/EBP-β, FAS, and ACC. (L–M) The phosphorylation of ERK1/2. (O–Q) Expression of GLUT4. All vaules are presented as the mean±standard error of the mean, n = 6. CUR, curcumin; ERK1/2, inhibited extracellular signal-regulated kinase 1/2; PPAR-γ, peroxisome proliferation-activity receptor-γ; C/EBP-β, CCAAT/enhancer-binding protein-β; FAS, fatty acid synthesis; ACC, acetyl-CoA carboxylase; GLUT4, glucose transporter type 4; DIM, differentiation induced media. * p<0.05 and ** p<0.01, compared with DIM cells; ^&&^ p<0.01, compared with DIM + CUR cells.

Numerous studies have suggested that ERK1/2 signaling pathway can function as a switch controlling adipogenesis by modulating PPAR-γ expression, therefore, we further elucidated ERK1/2 signaling pathways underlying PPAR-γ inhibition and anti-adipogenic effect of CUR in PSPA. Results showed that 20 μM CUR significantly decreased the mRNA and phosphorylation of ERK1/2 compared with DIM cells (Figure 4A–D), suggesting that ERK1/2 inhibition might contribute to CUR reduced lipid accumulation by inhibiting PPAR-γ expression.

**Figure 5:**
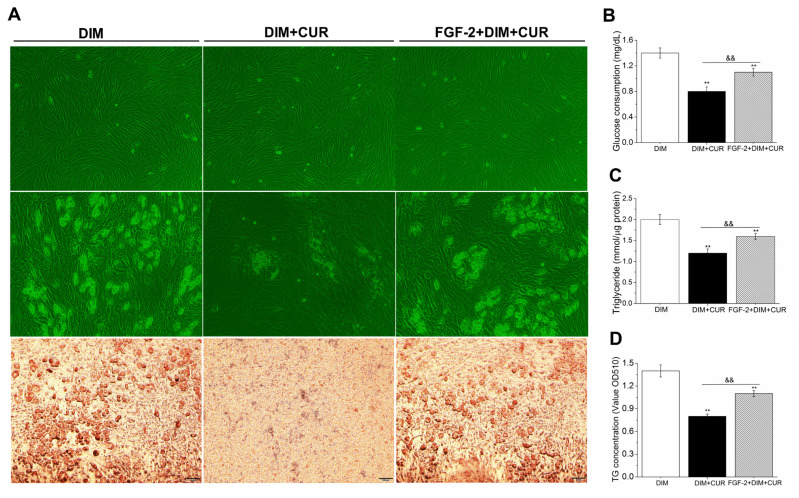
The reverse effect of ERK1/2 activation on CUR-reduced lipid accumulation and glucose consumption. (A) 85% to 90% confluence of PSPA, intracellular lipid accumulation and ORO staining (100×). (B) Glucose consumption. (C) ORO extracted and quantified by measuring the OD at 510 nm. (D): Intracellular TG content. All vaules are presented as the mean±standard error of the mean, n = 6. ERK1/2, inhibited extracellular signal-regulated kinase 1/2; CUR, curcumin; PSPA, porcine subcutaneous preadipocytes; ORO, oil red O; OD, optical density; TG, triglyceride; DIM, differentiation induced media. * p<0.05 and ** p<0.01, compared with DIM cells; ^&&^ p<0.01, compared with DIM + CUR cells.

Next, we sought to determine whether suppressed lipid accumulation by CUR could be reversed by ERK1/2 signaling pathway activation. The PSPA were pretreated with ERK1/2 activator fibroblast growth factor-2 (FGF-2) for 30 min to activate ERK1/2, and then were treated with CUR. Results showed that compared with DIM cells, the expression of ERK1/2, PPAR-γ, C/EBP-α, FAS, and ACC was decreased in CUR cells, while expression of these genes was significantly enhanced in FGF-2 pretreated cells compared with CUR alone cells (Figure 4E–M). In addition, both the mRNA and protein expression of GLUT4 (Figure 4N–P) were all significantly increased. In line with above results, increased lipid accumulation, higher positive rate of cytoplasmic ORO staining, elevated glucose comsumption and intracellular TG content (Figure 5A–D) were also observed in FGF-2 and CUR co-treated cells compared with CUR alone cells. Taken together, these above results further confirmed that the anti-adipogenic effect of CUR in PSPA was at least partially by repressing ERK1/2-PPAR-γ signaling pathway.

### Curcumin triggers apoptosis through increasing the Bax/Bcl-2 expression ratio and activating Caspase-3

**Figure 6:**
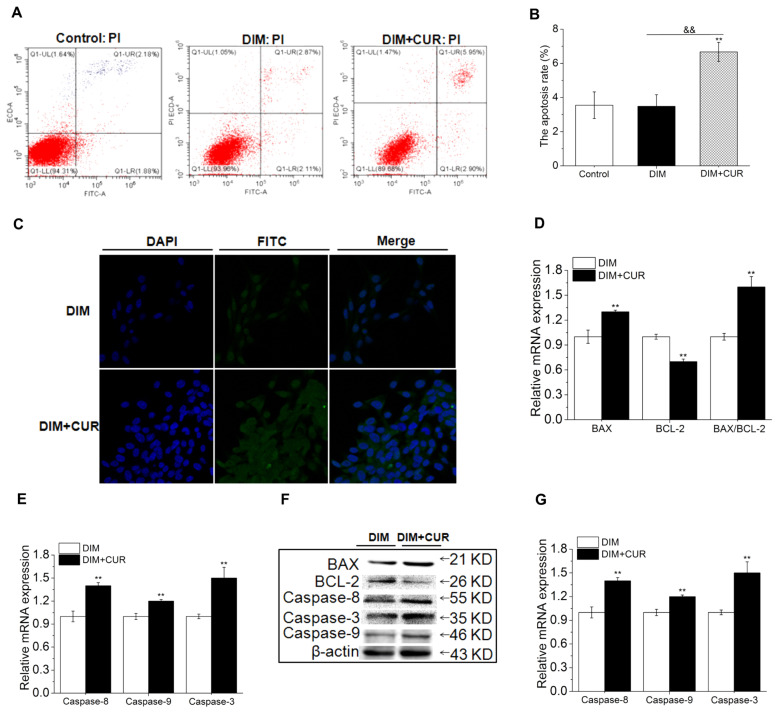
Effect of CUR on apoptosis signaling pathway. (A) Apoptosis detected by Flow Cytometry assay. (B) The apoptosis rate. (C) Apoptosis detected by TUNEL staining. (D) The mRNA expression of BCL-2 and BAX, and the BAX/BCL-2 ratio. (E) The mRNA expression of Caspase-3, -8 and -9. (F) The protein levels of BCL-2, BAX, Cleaved-Caspase-3, -8 and -9. All vaules are presented as the mean±standard error of the mean, n = 6. CUR, curcumin; DIM, differentiation induced media; TUNEL, terminal deoxynucleotidyl transferase-mediated deoxyuridine triphosphate-nick end labelling; BCL-2, B-cell lymphoma-2; BAX, BCL-2-associated X. * p<0.05 and ** p<0.01, compared with DIM cells; ^&&^ p<0.01, compared with DIM + CUR cells.

Adipocyte apoptosis has been proposed as a novel therapeutic strategy to reduce adipogenesis. Both flow cytometry and TUNEL methods were used to further detecte the mature adipocyte apoptosis. Based on the result of Figure 1, 30 μM CUR showed cytotoxicity on PSPA, therefore, only 30 μM CUR and DIM cells were used to determine the apoptosis rate and potential mechanisms. As shown in Figure 6A–D, 30 μM CUR significantly induced the apoptosis and upregulated the BAX mRNA expression as well as the BAX/BCL-2 expression ratio (162% higher than that of DIM cells), while decreased the mRNA expression of BCL-2 (antiapoptotic) compared with DIM cells.

The activation of Caspases has been shown to play crucial roles in the execution of apoptosis, therefore, the expression of Caspase-3, -8 and -9 was further determined. Results showed that 30 μM CUR significantly increased the mRNA expression of Caspase-3, -8 and -9 (Figure 6E). The protein levels of Cleaved Caspase-3, -8 and -9, as well as BCL-2 and BAX showed the same trends as the mRNA (Figure 6F, G), suggesting that the apoptosis was induced by increasing BAX/BCL-2 ratio and subsequently activating Caspase-3, -8 and -9. These data indicated that both extrinsic (Caspase-8) and intrinsic (Caspase-9) apoptotic pathways were involved in cytotoxic effects of CUR.

### AKT signaling pathway inactivation contrubuted to CUR reduced intracellular TG content and lipid accumulation by triggering apoptosis

**Figure 7:**
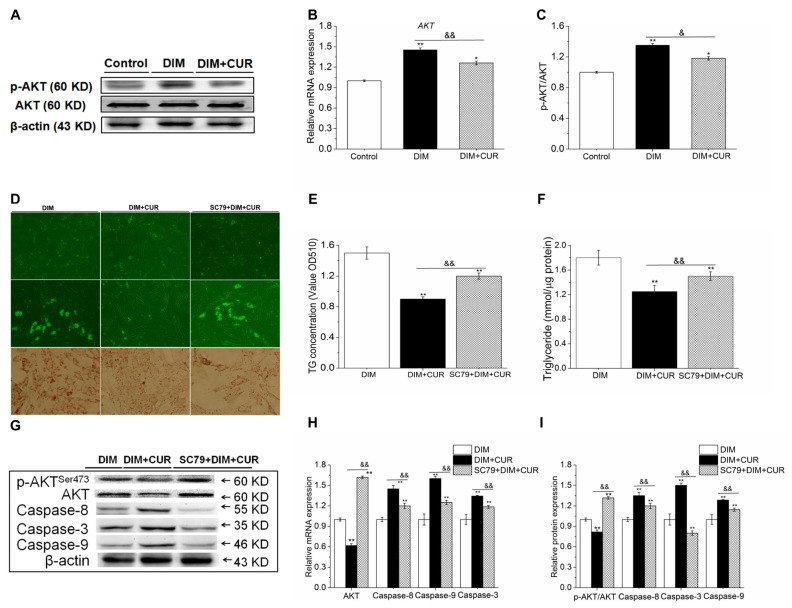
Effect of CUR on AKT signaling pathway and the reverse effect of AKT activation on CUR-induced apoptosis and lipid accumulation inhibition. (A) The mRNA expression of AKT. (B–C) The phosphorylation of AKT. (D) AKT activation on CUR-reduced lipid accumulation. (E) ORO extracted and quantified by measuring the OD at 510 nm. (F) Intracellular TG content. (G) AKT activation on mRNA expression of AKT, Caspase-3, -8 and -9. (H–I) AKT activation on protein levels of p-AKT, Cleaved-Caspase-3, -8 and -9. All vaules are presented as the mean±standard error of the mean, n = 6. CUR, curcumin; ORO, oil red O; OD, optical density; TG, triglyceride; DIM, differentiation induced media. * p<0.05 and ** p<0.01, compared with DIM cells; ^&^ p<0.05 and ^&&^ p<0.01, compared with DIM + CUR cells.

The Akt signaling pathway plays an important role in many cellular survival pathways, primarily as an inhibitor of apoptosis. To determine whether the cytotoxicity of CUR is dependent on AKT activity, PSPA were treated with CUR with or without an AKT activator (SC-79). Results showed that 30 μM CUR significantly reduced the mRNA expression and phosphorylation of AKT compared with DM cells (Figure 7A–C). Furthermore, compared with 30 μM CUR alone cells, CUR and SC79 in combination significantly attenuted CUR suppressed lipid accumulation (Figure 7D–H) by inhibiting apoptosis (Figure 7I, J). These results indicated that CUR activated apoptosis was partially mediated by repressing AKT signaling pathway and subsequently activating Caspase-3.

**Figure 8:**
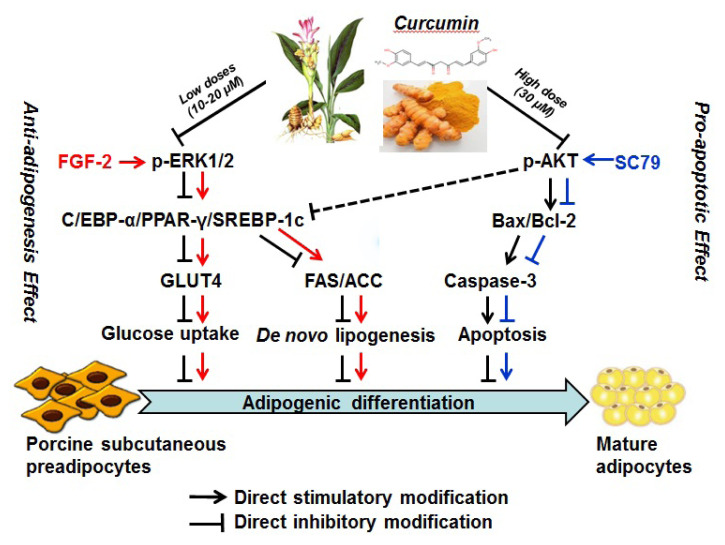
Proposed mechanism by which CUR inhibited lipid accumulation of PSPA. CUR inhibited adipogenic differentiation through repressing ERK1/2-PPAR-γ signaling pathway and subsequently inhibiting glucose uptake and *de novo* lipogenesis, and triggering apoptosis via inactivating AKT and increasing the BAX/BCL-2 ratio and Caspase-3 expression. CUR, curcumin; PSPA, porcine subcutaneous preadipocytes; ERK1/2, extracellular signal-regulated kinase 1/2; PPAR-γ, peroxisome proliferation-activity receptor-γ; BCL-2, B-cell lymphoma-2; BAX, BCL-2-associated X.

The fundamental mechanism by which CUR inhibited lipid accumulation was by inhibiting ERK1/2-PPAR-γ signaling pathway and triggering apoptosis in PSPA and is summarized in Figure 8.

## DISCUSSION

Subcutaneous (SAT), visceral, intermuscular and intramuscular fat (IMF), are the four types of fat depots that mainly exist in animal body. As the most important economic trait affecting meat quality, approaches for improving IMF content have focused on different meat animals. However, greater efforts should be made to clarify the deposition of SAT, since it is also an important trait directly affecting economic value and meat quality. Especially in pigs, SAT is the main fat storage type, however, its commercial value is extremely low. Excessive deposition of SAT will eventually lead to waste of feed and increase production costs in pig industry [17]. Furthermore, pigs have the strongest ability to deposit fat, especially the SAT, and serve as excellent models for studying metabolic diseases, since pigs and humans share high similarities in terms of physiological and metabolic characters [18]. As such, clarifying genes reducing SAT deposition benefits both animal production and human health disciplines.

Excessive fat accumulation is largely caused by increased number and/or expanded volume of adipocytes, therefore investigating adipocyte proliferation, differentiation and exploring the potential mechanisms are important approachs to control adipose tissue development [19]. Adipogenesis, the differentiation process of preadipocytes becoming mature adipocytes, is one of the most intensively studied models of cellular differentiation. Nowadays, researches have improved the knowledge of adipogenesis, mostly using the 3T3-L1 cell line, suggesting that 3T3-L1 can serve as a model for the adipogenic differentiation study [20–22]. However, it cannot be ignored that 3T3-L1 cells are a kind of immortalized murine cell, the adipogenesis in this cell line can not always represent that of other species. Therefore, in the present study, primary cultured PSPA were selected, mainly because that the biological characteristics of primary cells are much closer to the physiological state of the pig, and are more conducive to screen target genes in regulating adipogenesis. Furthermore, using PSPA as a model to study adipogenesis is much closer to the physiological process of adipocyte differentiation in the human body, and suggest that PSPA are the ideal cell model for obesity-related research. In addition, 3T3-L1 cells need incubation with cocktail of chemicals (insulin, dexamethasone, and IBMX) to differentiate into mature adipocytes. While, the PSPA have the advantages of not requiring such cocktail for the differentiation induction.

The CUR is an active ingredient extracted from the com monly-used spice turmeric plants and has been shown to have diverse biological functions. So far as we know, there is no report focusing on the effect of CUR on porcine lipid deposition. This *in vitro* study using PSPA provides the first evidence that CUR suppresses the lipid accumulation by both decreasing the adipocyte number and inhibiting adipogenic differentiation. Previous studies concluded in both 3T3-L1 mouse adipocytes and rat BMSCs also showed the negative role of CUR in adipogenic differentiation [9,23–25], which strongly supports our data. In addition, our unpublished data also revealed that CUR was able to improve the HFD-induced epididymal fat accumulation (data not shown). In addition, the lipids-lowering effect of CUR has been also proved in obese mice [26]. In a diabetic rat model, CUR reduces the contents of triglycerides, total cholesterol, blood glucose and free fatty acids [27]. These related *in vivo* investigations indirectly and strongly support our results. Furthermore, we revealed that the inhibitory role of CUR in proliferation was mediated by triggering apoptosis, whereas the decreased differentiation was mainly through inhibiting ERK1/2-PPAR-γ signaling pathway. These results are consistent with previous findings in differentiated 3T3-L1 cells that CUR is able to induce adipocyte apoptosis and suppress adipocyte differentiation, resulting in the inhibition of adipogenesis [25], and suggest that CUR is one of the most potent anti-obesity agents.

Numerous evidence has suggested that adipogenic dif ferentiation is a multi-step and complex biological process regulated by a series of transcription factors such as PPAR-γ and C/EBP-α [28]. Overexpression of PPAR-γ and C/EBP-α has been shown to promote adipogenesis, whereas PPAR-γ and/or C/EBP-α-deficient mice present lower fat mass [29]. In this study, we assumed that the inhibition of these two transcription factors and their target genes may participate in the anti-adipogenic effect of CUR on PSPA. Indeed, CUR significantly inhibited the transcriptional activity and expression of PPAR-γ and C/EBP-α, both of which have been well-considered as most important adipogenic genes and synergistically induce the adipocyte terminal differentiation [30]. In addition, both the expression of FAS and ACC, two direct target genes of PPAR-γ and C/EPB-α were also reduced significantly, suggesting that CUR significantly inhibited *de novo* lipogenesis. These results demonstrated that inhibition of PPAR-γ and subsequent repression of *de novo* lipogenesis can serve as the potential anti-obesity mechanism of CUR.

In our present study, we found that the glucose comsump tion and the expression of GLUT4 were both significantly suppressed, showing that CUR significantly inhibited the glucose uptake in adipocytes. The result coincided with the downregulation PPAR-γ and C/EBP-α, both of which are mainly induced in mature adipocytes and lead to the increased glucose uptake [31]. Our results suggested that the CUR reduced adipogenic differentiation and lipid accumulation of PSPA was closely related to glucose uptake inhibition. In addition, we also found that the expression of SREBP-1c was also obviously inhibited in CUR treated PSPA, and this can be partly explained by reduced PPAR-γ and C/EBP-α, both of which cross-regulated each other to directly regulate the target gene SREBP-1c. Furthermore, SREBP-1c inhibition also in turn decreased the FAS expression by inhibiting both PPAR-γ and C/EBP-α, since numerous evidence showed that SREBP-1c was able to stimulate the production of endogenous PPAR-γ ligand. The expression of Pref-1, a molecular switch of adipogenesis that acts by blocking adipocyte differentiation and keeping the preadipocyte state, was significantly increased in CUR treated PSPA compared with DIM cells, suggesting that CUR suppressed PSPA adipogenic differentiation by maintaining the preadipocyte state. The Pref-1 upregulation coincided with the decreased PPAR-γ and C/EBP-α, since previous study indicated that Pref-1 was able to block adipocyte differentiation through binding to the C/EBP-β promoter regions and preventing the activation of both C/EBP-α and PPAR-γ [32].

It is undoubted that PPAR-γ is necessary and sufficient to promote adipocyte differentiation [26]. Thus, an in-depth investigation to determine how CUR regulates the expression of PPAR-γ and its related adipogenic genes is in urgent need. Much evidence has suggested that ERK1/2 signaling plays a critical role in the process of cell proliferation and differentiation [27,33], and has proved that the ERK1/2 is implicated in adipogenesis through its downstream molecular target PPAR-γ [34]. In the current study, we found that CUR significantly inhibited the phosphorylation of ERK1/2 and subsequently suppressed the expression and trancriptional activity of PPAR-γ, providing the first evidence that CUR inhibited the PSPA adipogenic differentiation through inhibiting ERK1/2-PPAR-γ signaling pathways. This conclusion was strongly supported by the finding that ERK1/2 activation by FGF-2, an ERK1/2 activator, significantly abolished the inhibitory effects of CUR on the downregulation of PPAR-γ and C/EBP-α, and thus increased the glucose uptake, the *de novo* lipogenesis and lipid accumulation in PSPA. These results strongly suggested that the inhibition of ERK1/2-PPAR-γ signaling pathway, at least partially, contributed to the CUR suppressed glucose uptake and lipid accumulation, showing the positive role of ERK1/2 in promoting adipogenesis. However, some other studies have reached diametrically opposite conclusions, showing that the ERK1/2 activation regulated the adipocyte differentiation negatively via phosphorylation of PPAR-γ [35]. We postulate dual roles of ERK1/2 on adipogenic differentiation mainly because that the ERK1/2 is activated at different stages of adipocyte differentiation. In the other words, the ERK1/2 activation at the late stage of adipocyte differentiation inhibits the adipogenesis, while ERK1/2 activation during the early stage of adipocyte differentiation might promote it. The current results illustrated that CUR inhibited ERK1/2 phosphorylation and subsequent PPAR-γ suppression during the early stage might result in the negative effect on the PSPA differentiation. Our current results are also very similar to previous and reveal that Pref-1 upregulation is related to ERK1/2 inactivation and perform the anti-obesity effects in the HFD model.

Adipocyte number loss induced by apoptosis plays vital roles in strategies against obesity by regulating the number of adipocytes [36]. In current study, we demonstrated that adipocyte apoptosis was induced by CUR in PSPA. This is in accordance with previous result conducted in 3T3-L1 mouse adipocytes [25], indicating that CUR triggers adipocytes apoptosis regardless of the species involved. The present results also indicated that CUR induced apoptosis by activating Caspase-3, an important molecule participated in the final process of apoptosis. In addition, it is well known that the Bcl-2 family proteins play crucial roles in regulating apoptosis, and Bcl-2 is a negative regulator of cell apoptosis [37]. However, the Bax is a proapoptotic protein, which exists in the mitochondrial outer membrane and translocates into the mitochondria during early apoptosis, inducing apoptosis signal transduction. The present findings showed that CUR upregulated the expression of Bax while down regulated the Bcl-2, leading to an increase of the Bax/Bcl-2 expression ratio. These results suggest that the alterations of Bcl-2 family proteins largely contribute to CUR inducing apoptosis. It is known that mitochondria cytochrome-c release is therefore considered a key step in the initial apoptotic process, and its release is regulated by numerous Bcl-2 family proteins [38]. Therefore, in our following study, cytochrome-c should be further determined to further explore the relationship between Bcl-2 family and Caspase-3. In conclusion, the present results suggest that the Bcl-2 family proteins and Caspase-3 alterations are responsible for CUR induced adipocyte mitochondrial apoptotic pathway in PSPA. In addition, in this study, we found that CUR significantly inhibited the AKT phosphorylation, while pretreatment with SC-79, a chemical activator of AKT significantly increased the AKT and completely reversed the apoptosis and the inhibitory effect on lipid accumulation of CUR, suggesting anti-apoptotic effect of AKT on apoptosis. This was consistent with numerous previous studies which clearly demonstrated that Akt exhibits an anti-apoptotic role in most contexts [39,40]. However, several studies have been reported that Akt pathway also exerts proapoptotic effects in response to various stimuli and plays essential roles in adipogenesis [41], and more studies are needed to clarify this difference. These data collectively revealed that CUR may be a promising therapeutic agent against obesity, by increasing the apoptosis induced adipocyte number loss.

## CONCLUSION

Taken together, the study was aimed to explore the effect of CUR on lipid accumulation in PSPA, and to further determine the potential mechanisms, and current results provides the first evidence that CUR inhibits adipogenic differentiation and lipid accumulation of PSPA, partly by inhibiting ERK1/2-PPAR-γ signaling pathway and subsequently suppressing the glucose uptake and *de novo* lipogenesis. Additionally, the apoptosis pathway was triggered by inactivating AKT and increasing BAX/BCL-2 ratio and Caspase-3, and contributed to CUR-repressed lipid accumulation (Figure 8). These new findings provide a novel mechanism by which CUR effectively inhibits the lipid accumulation of PSPA, and an lead to CUR being developed as a natural and effective agent for the reduction of porcine fat deposition, as well as the prevention and treatment of human obesity.

## Figures and Tables

**Table 1:** The primer sequences used for real-time quantitative polymerase chain reaction

Gene	GenBank accession No.	Primer sequence (5′to 3′)
*PPAR-γ*	NM_138711	F: GCCCTTCACCACTGTTGATT
		R: GAGTTGGAAGGCTCTTCGTG
*C/EBP-β*	NM_001199889	F: GACAAGCACAGCGACGAGTA
		R: AGCTGCTCCACCTTCTTCTG
*FAS*	NM_213839.1	F: GTCCTGCTGAAGCCTAACTC
		R: TCCTTGGAACCGTCTGTG
*ACC*	NM_133360.2	F: GGCCATCAAGGACTTCAACC
		R: ACGATGTAAGCGCCGAACTT
*SREBP1c*	NM_214157.1	F: TTCTGGAGACATCGCAAACAA
		R:TGGTAGACAACAGCCGCATC
*C/EBP-α*	XM_003127015.4	F:CAAAGCCAAGAAGTCGGTAGA
		R: ATTGTCACTGGTCAGCTCCA
*AP2*	NM_001002817.1	F: GAGCACCATAACCTTAGATGGA
		R: AAATTCTGGTAGCCGTGACA
*GLUT4*	NM_001128433.1	F: GCTGCCTCCTACGAGATGCT
		R: TGGCCAGCTGGTTGAGTGT
*Pref-1*	NM_001048187.1	F: CCCATGGAGCTGAATGCCT
		R: TTGCAAATGCACTGCCAGGG
*PPIA*	NM_214353.1	F:TCCTCCTTGGTGCTAATCTCGT
		R:TGATCTTCTTGCTGGTCTT

*PPAR-γ*, peroxisome proliferation-activity receptor-γ; *C/EBP-β*, CCAAT/enhancer-binding protein-β; *FAS*, fatty acid synthesis; *ACC*, acetyl-CoA carboxylase; *SREBP1c*, sterol-regulatory element binding protein 1c; *AP2*, adipocyte protein 2; *GLUT4*, glucose transporter type 4; *Pref-1*, preadipocyte factor-1; *PPIA*, peptidylprolyl isomerase A.

**Table 2:** The antibodies used in the present study

Antibody	Introduction and company	Purpose
PPAR-γ	Polyclonal rabbit antibody, 1:1,000, AP0686, Bioworld Technology, USA	Analysis of PPAR-γ
C/EBP-α	Polyclonal rabbit antibody, 1:1,000, BS1384, Bioworld Technology, USA	Analysis of C/EBP-α
SREBP-1c	Polyclonal rabbit antibody, 1:1,000, BS70008, Bioworld Technology, USA	Analysis of SREBP-1c
AP2	Polyclonal rabbit antibody, 1:1,000, #3208, Cell Signaling, USA	Analysis of AP2
FAS	Polyclonal rabbit antibody, 1:500, 13098-1-AP, Proteintech™, USA	Analysis of FAS
ACC	Rabbit polyclonal antibody, 1:500, BS90018, Proteintech™, USA	Analysis of ACC
GLUT4	Polyclonal rabbit antibody, 1:1,000, BS3680, Bioworld Technology, USA	Analysis of GLUT4
AKT (Ser473)	Rabbit polyclonal antibody, 1:500, Cat. No. A00959, GenScript, USA	Analysis of AKT
Phosphorylated AKT (Phospho-Ser473)	Rabbit polyclonal antibody, 1: 500, Cat. No. A00965, GenScript, USA	Analysis of phosphorylated AKT
ERK1/2	Rabbit polyclonal antibody, 1:500, #9101, Cell Signaling, USA	Analysis of ERK1/2
Phosphorylated ERK1/2	Rabbit polyclonal antibody, 1:500, #4695, Cell Signaling, USA	Analysis of phosphorylated ERK1/2
BAX	Rabbit polyclonal antibody, 1:500, BS90120, Bioworld Technology, USA	Analysis of BAX
BCL-2	Rabbit polyclonal antibody, 1:1,000, BS70205, Bioworld Technology, USA	Analysis of BCL-2
Caspase-8	Rabbit polyclonal antibody, 1:1,000, BS90191, Bioworld Technology, USA	Analysis of Cleaved-Caspase-8
Caspase-3	Rabbit polyclonal antibody, 1:1,000, BS90181, Bioworld Technology, USA	Analysis of Cleaved-Caspase-3
Caspase-9	Rabbit polyclonal antibody, 1:1,000, BS90192, Bioworld Technology, USA	Analysis of Cleaved-Caspase-9
β-actin	Mouse monoclonal antibody, 1:1,0000, sc-130656, Santa Cruz, USA	Analysis of β-actin

PPAR-γ, peroxisome proliferation-activity receptor-γ; C/EBP-α, CCAAT/enhancer-binding protein-α; SREBP1c, sterol-regulatory element binding protein 1c; AP2, adipocyte protein 2; FAS, fatty acid synthesis; ACC, acetyl-CoA carboxylase; GLUT4, glucose transporter type 4; ERK1/2, extracellular signal-regulated kinase 1/2; BAX, BCL-2-associated X; BCL-2, B-cell lymphoma-2.
